# The emergence of cohorts of co-active neurons in random recurrent networks provides a mechanism for orientation and direction selectivity

**DOI:** 10.1186/1471-2202-15-S1-P129

**Published:** 2014-07-21

**Authors:** Dmitry Tsigankov, Matthias Kaschube

**Affiliations:** 1 Frankfurt Institute for Advanced Studies, Frankfurt, Germany

## 

We study random strongly heterogeneous recurrent networks of firing rate neurons, introducing the notion of cohorts: groups of co-active neurons, who compete for firing with one another and whose presence depends sensitively on the structure of the input. The identities of neurons recruited to and dropped from an active cohort changes smoothly with varying input features. We search for network parameter regimes in which the activation of cohorts is robust yet easily switchable by the external input and which exhibit large repertoires of different cohorts. We apply these networks to model the emergence of orientation and direction selectivity in visual cortex. We feed these random networks with a set of harmonic inputs that vary across neurons only in their temporal phase, mimicking the feedforward drive due to a moving grating stimulus. The relationship between the phases that carries the information about the orientation of the stimulus determines which cohort of neurons is activated. As a result the individual neurons acquire non-monotonic orientation tuning curves which are characterized by high orientation and direction selectivity. This mechanism of emergence for direction selectivity differs from the classical motion detector scheme, which is based on the nonlinear summation of the time-shifted inputs. In our model these two mechanisms coexist in the same network, but can be distinguished by their different frequency and contrast dependences. In general, the mechanism we are studying here converts temporal phase sequence into population activity and could therefore be used to extract and represent also various other relevant stimulus features.

